# Acromion and Clavicle Stress Fractures After Reverse Total Shoulder Arthroplasty Reflect Failure to Address Osteoporosis: A Case Report and Literature Review

**DOI:** 10.7759/cureus.79993

**Published:** 2025-03-03

**Authors:** John T Cronin, Kevin B Curtis, Brett W Richards, Julia N Hibbard, John G Skedros

**Affiliations:** 1 Shoulder & Elbow, Utah Orthopaedic Specialists, Salt Lake City, USA

**Keywords:** acromion fracture, clavicle fracture, osteoporosis, reverse shoulder arthroplasty, shoulder replacement, stress fracture

## Abstract

The exponential increase in the rate of reverse total shoulder arthroplasty (RTSA) has been accompanied by a rise in complication rates of this procedure. Of these, peri-prosthetic stress fractures can be particularly problematic due to their potential to cause significant impairment of shoulder function. Despite the association between these stress fractures and osteopenia/osteoporosis, pre-operative bone density assessment is not standard practice for elective RTSA. We report the case of a 68-year-old female patient who, at eleven weeks after elective RTSA (for rotator cuff-tear arthropathy), experienced a non-traumatic stress (insufficiency) fracture of the acromion process of the ipsilateral scapula. Thirteen weeks later, new-onset pain occurred with minimal shoulder use, and a midshaft clavicle stress fracture was detected. She was then diagnosed and treated for osteoporosis, vitamin D deficiency, and hypothyroidism. An ultrasound-based bone-growth stimulator was used to treat both fractures, but only the acromion fracture healed. The clavicle fracture became a 100% displaced chronic non-union. However, the patient felt that surgical fixation of the clavicle fracture would not provide a significant benefit. At 1.5 years after the RTSA, she was moderately satisfied with her shoulder function and highly satisfied with pain reduction, and no additional surgery was required. This is the first reported case describing a patient with acromion and clavicle stress fractures occurring in association with ipsilateral RTSA. We also review the literature of cases with clavicle stress fractures in association with RTSA and highlight key findings: (i) the prevalence of osteoporosis in the population undergoing shoulder arthroplasty is high and (ii) performing shoulder arthroplasty on patients with poor bone quality presents multiple challenges that are underappreciated. This case underscores the importance of pre-operative bone density/health screening to mitigate stress fracture risk after RTSA.

## Introduction

Many complications have been reported in association with reverse total shoulder arthroplasty (RTSA) [1-3]. Among these, stress fractures are common, with acromion and scapular spine fractures being the most frequently observed [4]. The incidence of acromion and scapular spine stress fractures following RTSA typically ranges from 0.8 to 11.2% of total patients [1,3,5-12]. In contrast, stress fractures of the clavicle in association with RTSA are rare, as we located only five reports of this association [13-17]. Notably, we found no documented cases of concurrent acromion and clavicle stress fractures occurring in association with ipsilateral RTSA. In the patient reported here, acromion and clavicle stress fractures occurred non-synchronously within three months of ipsilateral RTSA. A conservative treatment approach with a bone-growth stimulator was used to treat our patient’s fractures; this modality is gaining popularity in managing stress fractures of the shoulder region following RTSA [9,14,18]. However, in our patient only the acromion fracture was successfully treated with the use of an ultrasound-based bone-growth stimulator.

Definition of "stress fracture" and different categorizations for stress fractures

Stress fractures are traditionally classified into three types: fatigue fractures due to overuse, insufficiency fractures due to bone fragility, and pathologic fractures due to bone weakness involving tumors [19,20]. Fatigue fractures occur in normal bone that has been overused, such as in military personnel and athletes, whereas insufficiency fractures develop in fragile bone that is repeatedly subjected to low levels of stress during routine activities [20]. In this case report, we refer to stress fractures as “insufficiency fractures,” a term commonly used in studies involving shoulder arthroplasty [1,5,21]. It should be noted that nearly all studies that have reported on stress fractures in association with RTSA describe them as “periprosthetic.” However, we found one study that differentiated stress fractures that occur in association with RTSA from periprosthetic fractures [1]. Our extensive literature search for clavicle and acromion stress fractures in association with RTSA included all relevant studies, regardless of how the fractures were defined or categorized.

We report this unique case and review the literature of cases with clavicle stress fractures in association with RTSA. This case also underscores the importance of preoperative bone density and associated health screenings to reduce the risk of stress fractures in the shoulder region after RTSA.

## Case presentation

**Figure 1:**
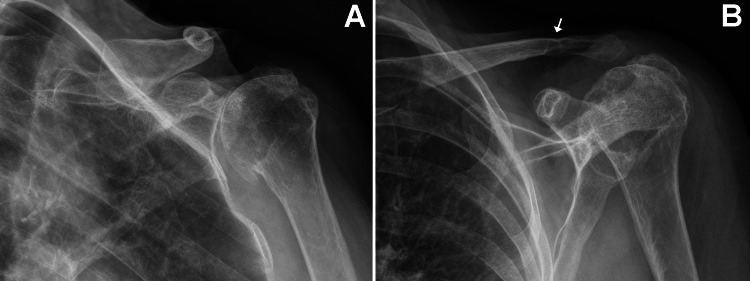
Preoperative radiographs of the patient’s left shoulder showing end-stage rotator cuff-tear arthropathy (A) Anterior-posterior view; (B) oblique scapula Y view. The site of the prior clavicle fracture has patchy lucencies (arrow) that suggest compromised bone density. Generalized osteoporosis is evident in these radiographs (reduced bone density, thin cortices).

**Figure 2:**
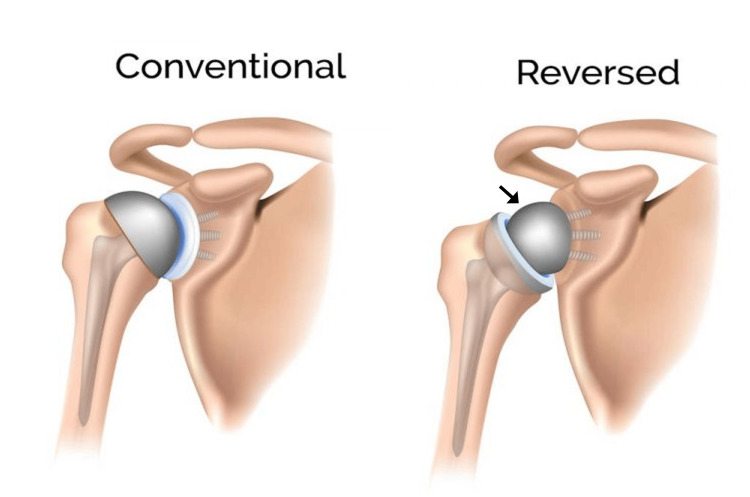
Conventional vs. reverse (reversed) total shoulder prostheses At left, conventional (anatomic) total shoulder prosthesis where the spherical portion is at the location of the natural/anatomic humeral head (which is resected and replaced). At right, reverse total shoulder prosthesis where the spherical portion is at the glenoid (socket portion of shoulder) and is known as the “glenosphere” (arrow). Source: [22] Reproduced with permission from the Bangalore Shoulder Institute

**Figure 3:**
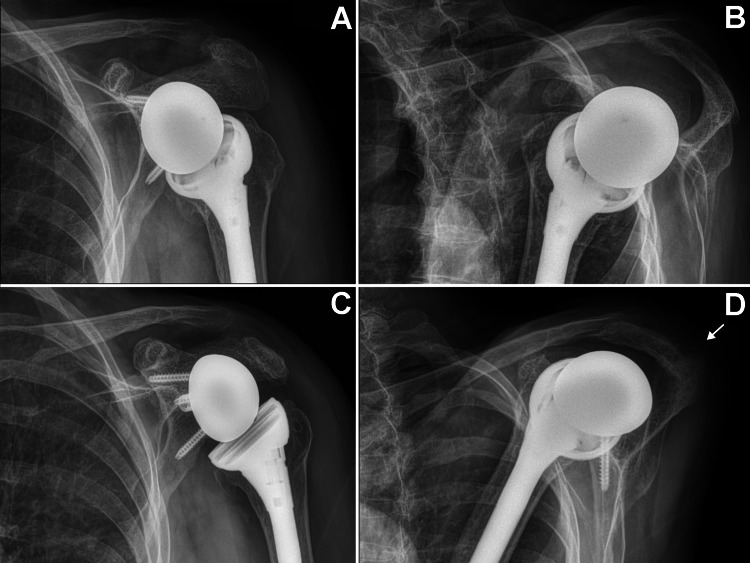
Radiographs taken three weeks (A, B) and four months (C, D) after RTSA A and B: Radiographs taken at three weeks after the RTSA (no complications had occurred). C and D: Radiographs taken at four months after the RTSA showing an angulated acromion stress fracture (arrow) that narrows the subacromial space. There was no pain with palpation of the clavicle.  (A and C) Anterior-posterior views and (B and D) scapula Y views. RTSA: Reverse total shoulder arthroplasty

A 68-year-old (155 cm tall, 55 kg; BMI = 23) left-hand dominant woman presented with end-stage rotator cuff-tear arthropathy (Figure 1) that was treated with RTSA. Figure 2 shows the main differences between an anatomic TSA and RTSA. The patient’s RTSA was performed on August 22, 2023, by JGS. The prosthesis was a DePuy DELTA XTEND® (Depuy Synthes, Warshaw, Indiana, USA) and included these components: 10mm press-fit humeral stem with a neck-shaft angle of 145° (where 180° is vertical), 3mm-thick polyethylene liner, standard glenoid baseplate, and 38mm diameter glenosphere with 2mm lateral offset (Figure 3). There were no perioperative complications.

Twenty years prior to surgery, she sustained a closed ipsilateral clavicle shaft fracture, which was treated non-operatively. At the time of the RTSA, she had a 15-year history of smoking cigarettes and occasional alcohol consumption. Her medical history included depression, bipolar disorder, and a stroke four years earlier, though without any notable lasting effects. Before surgery, her regular medications included atorvastatin calcium (80 mg/day), citalopram hydrobromide (20 mg/day), Iamotrigine (25 mg/day), and a nicotine transdermal patch (14 mg/24hr). Prior to surgery, there had been no workup for the status of her bone health/density (e.g., vitamin D deficiency, osteoporosis, etc.).

Eleven weeks after surgery, the patient noted new-onset left shoulder pain localized over the basilar acromion region. The pain began following activities such as lifting grandchildren, doing laundry, carpet vacuuming, and overhead lifting of objects weighing 1-5 kg. Radiographs showed an angulated fracture near the base of the acromion (Figures 3C, 3D). She was instructed to wear a sling for six weeks, a commonly recommended treatment for this type of fracture [11,21,23,24]. If healing did not show sufficient progress by 90 days after fracture, the plan was to use a bone-growth stimulator, as per USA Medicare criteria [25].

**Figure 4:**
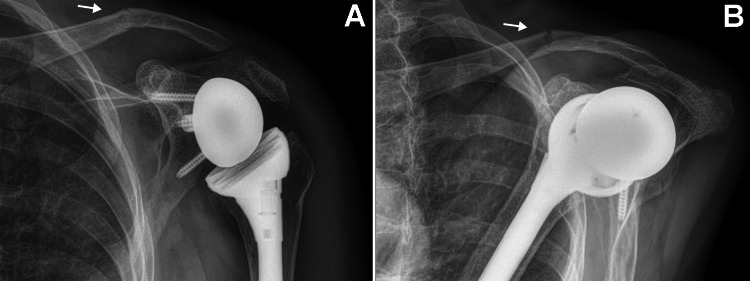
Radiographs taken six months after RTSA. Radiographs of the left shoulder taken six months after the RTSA showing a mildly angulated fracture of the clavicle (arrows) in addition to the angulated fracture of the acromion. The clavicle fracture is in the vicinity of the prior (healed) clavicle fracture, as seen in Figure 1. (A) Anterior-posterior view and (B) scapula Y view. RTSA: Reverse total shoulder arthroplasty

At 24 weeks after surgery (February 7, 2024), the patient presented with complaints of acute new-onset pain in the left clavicle region that began after she rolled onto that shoulder while sleeping. Radiographs showed a non-displaced midshaft clavicle fracture, in addition to the existing acromion stress fracture that showed delayed healing (and was also painful to palpation) (Figure 4). The new clavicle fracture occurred in the vicinity of the fracture that had occurred many years prior, which likely resulted in compromised bone density as suggested by findings in Figure 1B. At the time of the new clavicle fracture, a bone-growth stimulator had not yet been used for the acromion fracture.

At approximately 100 days after the acromion fracture, treatment with a bone-growth stimulator (Exogen®, Bioventus LLC, Durham, North Carolina, USA) was initiated. At a follow-up visit 28 weeks after the RTSA and four weeks after the clavicle fracture (March 7, 2024), the clavicle fracture was found to be 100% displaced. She denied any trauma that might have caused this displacement and declined our recommendation for surgical reconstruction. To enhance healing of the clavicle fracture, we instructed her to use the same bone-growth stimulator that was being used for the acromion fracture.

The patient subsequently underwent workup for osteoporosis with a dual-energy X-ray absorptiometry (DEXA) scan and blood testing. The results are shown in Table 1 and Table 2. They revealed subclinical hypothyroidism and osteoporosis. Risks of major osteoporotic fractures were then calculated using the "fracture risk assessment tool" (FRAX score), which revealed a risk of 26% for major osteoporotic fracture and 11% for hip fracture. FRAX is a widely accepted tool for fracture risk assessment that uses clinical risk factors optionally combined with femoral neck BMD to estimate the 10-year probability of major osteoporotic fracture (hip, clinical spine, humerus or wrist fracture) and the 10-year probability of hip fracture [26,27]. A FRAX calculated risk of ≥20% for a major fracture and/or ≥3% for a hip fracture are considered high risk and the threshold for clinical intervention [28].

**Table 1:** Bone density (DEXA)* results. *All of the T-scores shown above are indicative of osteoporosis (i.e., T score < -2.5). DEXA: Dual-energy X-ray absorptiometry

Location	T Score*
Lumbar spine (L1-L2)	-3.2
Left femoral neck	-2.7
Right femoral neck	-2.7

**Table 2:** Blood test results. TSH: Thyroid stimulating hormone; CMP: comprehensive metabolic panel; ALP: alkaline phosphatase

Parameter	Result	Reference Range
25-hydroxyvitamin D	Low (24 ng/mL)	Normal range: 30-80 ng/mL
Calcium	Normal	
TSH	High (11.5 uIU/mL)	Normal range: 0.27-4.20 uIU/mL
Thyroxine (T4)	Normal	
CMP	Normal, except for high ALP (111.0 U/L)	ALP normal range: 35-104 U/L

The patient was referred to her primary care provider for additional workup and treatment of her osteoporosis and subclinical hypothyroidism. Additional bone metabolism markers were not measured. Although an anabolic/osteogenic agent (e.g., teriparatide) was recommended, her health insurance company denied coverage due to its relatively high cost. Instead, at five months after commencing the use of the bone-growth stimulator, she was started on a weekly oral dose of a bisphosphonate (alendronate 70 mg).

**Figure 5:**
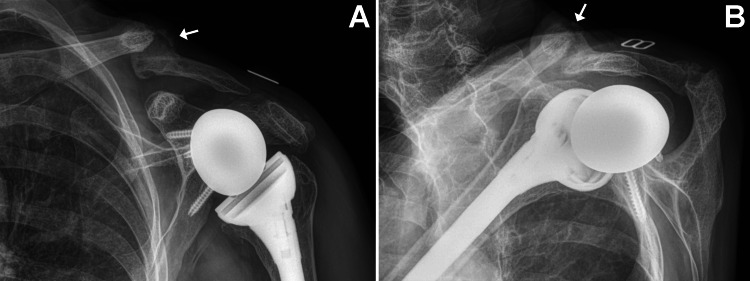
Radiographs taken 13 months after RTSA. Radiographs of the left shoulder taken 13 months after the RTSA showing evidence of healing of the acromion fracture and signs of healing at the site of the clavicle fracture, which had become 100% displaced (arrows). There was mild pain with palpation of the clavicle fracture site, but no pain with palpation of the acromion fracture site. (A) Anterior-posterior view and (B) scapula Y view. RTSA: Reverse total shoulder arthroplasty

Eight weeks after initiating treatment with the bone-growth stimulator, radiographs showed healing of the acromion fracture and what appeared to be improved callus formation at the clavicle fracture site (Figure 5). Radiographs taken two months later confirmed healing of the acromion fracture but showed that the clavicle fracture had not healed. The stimulator was used for one additional month (five months total) at each fracture site. At a follow-up clinic visit at seven months after the clavicle fracture, she reported that the pain in the region of the clavicle was typically minimal, but pain increased to moderate when attempting to participate in her favorite sport, pickleball (a tennis-like sport). She scored 44 on the Disabilities of the Arm, Shoulder, and Hand (DASH) questionnaire, which indicated moderate disability (best score = 0, worst score = 100; moderate disability: 16-45) [29,30].

**Figure 6:**
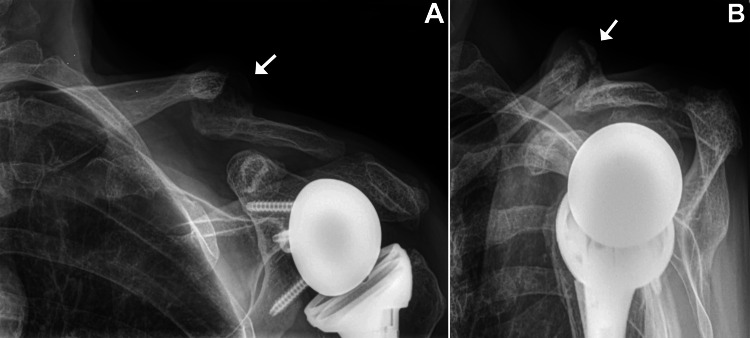
Radiographs taken 18 months after RTSA. Radiographs of the left shoulder taken 18 months after the RTSA (12 months after the occurrence of the clavicle fracture). (A) Anterior-posterior view and (B) scapula Y view. Arrows indicate the clavicle fracture non-union. RTSA: Reverse total shoulder arthroplasty

**Figure 7:**
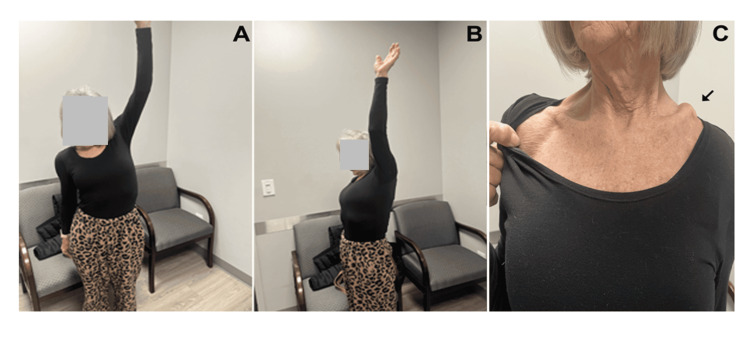
Photographs at 18 months after RTSA. Photographs showing maximum active motion of her shoulder in abduction (A) and flexion (B). The location of the left clavicle fracture non-union can be seen in C (arrow); the right clavicle is completely normal. RTSA: Reverse total shoulder arthroplasty

At the final follow-up, 13 months after the clavicle fracture and 19 months after RTSA, radiographs showed a non-union of the left midshaft clavicle fracture and a well-healed left acromion fracture (Figure 6). At that clinic visit, her shoulder ranges of motion were as follows: 160° forward flexion, 125° forward abduction, 60° external rotation, and 50° internal rotation (Figure 7).

The patient declined surgical fixation because she had no pain with most activities of daily living. When pain occurred, it was usually <2/10 (10 being the worst) and usually only occurred when performing activities such as mopping floors or repetitive overhead reaching. Her DASH score improved to 32; the main reason for this intermediate score reflected her inability to participate in pickleball and related activities. Overall, she was moderately satisfied with her function and highly satisfied with her pain reduction. 

## Discussion

The use of RTSA has increased dramatically in the United States, with a nearly three-fold increase from 2012 to 2017, and it has become the most common method of shoulder arthroplasty in the United States [31-33]. While RTSAs are now an established treatment option for patients with rotator cuff-tear arthropathy or large, irreparable rotator cuff tears, RTSAs are associated with various complications. For example, in a review of 78 studies reporting on 4,124 RTSA procedures, Bohsali et al. [1] reported an overall complication rate of 16.1%. As a percentage of the total number of these complications, specific complications included glenohumeral instability (31.3%), infection (17.8%), component loosening (11.3%), neural injury (7.5%), post-operative periprosthetic fractures (6.6%; typically fractures of the humerus and glenoid regions), and acromion and/or scapular spine stress fractures (6.0%). As a percentage of all 4,124 RTSA procedures, the incidence of post-operative periprosthetic fractures was 1.1%, and the incidence of stress fractures of the acromion and scapular spine was 1.0%. Although their literature search included studies published from 2006 to 2015, they did not locate any study reporting a clavicle stress fracture in association with RTSA (note that Anakwenze et al. was published in 2014). This demonstrates the rarity of our patient’s case.

We located one case of a spontaneous clavicle fracture occurring in a patient with poor quality bone, without any traumatic event or history of prior or concurrent shoulder arthroplasty [34]. Clavicle stress fractures have also been reported in other patients without predisposing risk factors, traumatic events, or prior shoulder arthroplasty [35,36]. We located five case reports of clavicle stress fractures occurring after RTSA [13-17]. The treatment and outcomes of the patients in these cases are summarized in Table 3. It should be noted that the patient in Streck et al. [16] suffered from multiple pre-existing conditions, including rheumatoid arthritis and advanced Parkinson’s disease with recurrent falls. Additionally, she had osteoporosis following long-term glucocorticoid and methotrexate treatment, with multiple pathological fractures for nearly 15 years before the RTSA. For nearly eight years before the RTSA, she had been receiving denosumab, an injectable non-bisphosphonate medication that reduces bone resorption [37].

**Table 3:** Review of cases with clavicle stress fractures on the same side (ipsilateral) of RTSA. ORIF: Open reduction internal fixation; F: female; RTSA: reverse total shoulder arthroplasty

Author	Age	Sex	Indications for RTSA	Time of Fracture Occurrence after RTSA	Treatment	Outcome
Anakwenze et al. [13]	82	F	Rotator cuff-tear arthropathy	10 weeks	10 weeks of conservative treatment without the use of a bone-growth stimulator, followed by ORIF	Satisfactory
Kim et al. [14]	64	F	Irreparable rotator cuff tear	3 weeks	Conservative treatment without the use of a bone-growth stimulator, consisting of immobilization, pendulum exercises, and passive motion	Fracture healed
Mufty et al. [15]	90	F	Irreparable rotator cuff tear	10 months	Conservative treatment without the use of a bone-growth stimulator, consisting of a figure-of-eight bandage; ORIF was suggested at 12 months after her RTSA, but the patient was reluctant to undergo surgery because of her age and comorbidities	Pain symptoms moderately improved; abduction and forward flexion each <90°; persistent nonunion
Streck et al. [16]	69	F	Rotator cuff-tear arthropathy	18 weeks	ORIF which failed after five weeks due to screw breakage and fracture displacement, though no trauma had occurred; due to the patient’s medical problems, relatively poor bone quality, and thrombocytopenia, no further surgical treatment was done	Three months after ORIF, the patient had significant improvement in pain, though with severely limited shoulder motion; persistent nonunion
Yoshida and Yoshida [17]	87	F	Irreparable rotator cuff tear	6 months	Conservative treatment without the use of a bone-growth stimulator, consisting of sling immobilization for three weeks, followed by graded passive range of motion exercises within a tolerable range	At three months post-fracture, pain relieved; six months post-fracture, complete union and pre-fracture range of motion achieved

In their recent case report describing an 87-year-old female patient with a distal clavicle stress fracture at six months after RTSA (caused by pulling on a dog leash during a routine walk), Yoshida and Yoshida [17] overview the literature regarding potential mechanisms of this complication. Notably, patients undergoing RTSA experience an increase in the arm length [38], which increases tension and loading on the deltoid muscle origins due to the elongated lever arm arising from the medialization of the center of rotation [39]. Consequently, fractures of the deltoid muscle origin may occur as a result of over-tensioning or lateralization of the deltoid, leading to excessive bone strain (tissue deformation) in these regions. The middle-to-posterior belly of the deltoid muscle originates from the acromion and scapular spine, where stress fractures have been reported in association with RTSA in up to 7.2 % of cases [40]. Although the anterior belly of the deltoid muscle originates from both the lateral one-third of the clavicle and the anterior acromion, fractures occur mostly at the acromion. This is likely a consequence of several inter-related factors, including (i) in RTSA, there is inherent change in upper limb biomechanics that results in increased stresses on the acromion and scapular spine likely to a much greater extent than on the clavicle, (ii) the deltoid muscle generally exerts more stress on acromion and lateral scapular spine than on the clavicle, as a larger portion of the deltoid muscle originates from these two regions when compared to the clavicle, and (iii) the overall contribution of the deltoid to abduction strength (mainly involving the acromion region) is higher than to flexion strength (mainly involving the clavicle region) (see [41] and review of biomechanical studies in [39]).

We treated our patient’s acute midshaft clavicle fracture with a bone-growth stimulator despite the lack of clear evidence supporting its efficacy in enhancing or accelerating healing for acute fractures [42]. However, bone-growth stimulators are well-established as a viable non-invasive treatment option for various fracture non-unions [43-46]. These devices function via ultrasound or electrical stimulation applied on the skin surface, overlaying the non-union site. We used an ultrasound-based stimulator (Exogen®) that emits low intensity, pulsed ultrasound (LIPUS) that induces angiogenesis, osteogenesis, bone mineralization, and matrix remodeling [43].

Stimulators emitting LIPUS have been used to treat various fracture non-unions, with success rates ranging from 68 to 96% [35-38]. The overall average success rate of >80% when using the LIPUS devices in treating fracture non-unions is comparable to the success rate of surgical treatment of non-infected non-unions [44]. In general, these devices are also much less expensive than surgical treatments for fracture non-unions [47,48]. Although there are robust studies showing high success rates of bone-growth stimulators in treating various fracture non-unions, their specific use in treating stress fractures associated with RTSA has only been reported anecdotally [9,18,49]. Controlled prospective studies are needed to establish the efficacy of this method conclusively when compared to conservative treatment involving only the use of a sling.

Our patient accepted non-operative management of her clavicle fracture non-union. For surgeons and other healthcare providers who might be faced with a patient with a displaced clavicle fracture on the same side of RTSA, it is useful to consider the literature regarding the typical management of such fractures. Althausen et al. [50] performed a retrospective study of a cohort of 204 adult patients who were treated for isolated, displaced, and closed midshaft clavicle fractures. Of these, 149 met the study criteria, with 66 treated non-operatively and 83 treated with open reduction internal fixation using a dorsal plate and screws. None of the fractures were reported to be in chronologic proximity of an ipsilateral shoulder arthroplasty. There were no significant differences between the groups in terms of age, sex distribution, handedness, tobacco use, diabetes, radiographic displacement (~40-42 mm), or participation in manual labor. However, the operative group had significantly greater satisfaction with the outcome and significantly lower chronic pain, cosmetic deformity, weakness, loss of motion, and non-union (0% in operative vs. 4.8% in non-operative). These results echo the clear clinical effectiveness of surgical stabilization of displaced midshaft clavicle fractures in adults [51-57]. Other authors have shown non-union rates of up to 15% and report a 25% decrease in shoulder and arm strength in non-operatively managed adult patients with displaced midshaft clavicle fractures [58,59]. In a meta-analysis of randomized clinical trials comparing non-operative and operative treatment for displaced midshaft fractures in adults, McKee et al. [51] reported an overall non-union rate of 15% for nonoperative treatment versus 1% for operative treatment across all included studies. Despite this discrepancy, the functional outcome of healed fractures was similar in both groups, suggesting that the primary advantage of surgical fixation results mainly from the prevention of non-unions [60,61].

Although operative management of displaced midshaft clavicle fractures generally results in more favorable outcomes than non-operative treatment, our patient achieved a good outcome despite the non-operative management of her non-union. She belonged to the small subset of patients who can successfully avoid surgery due to lower daily physical demands [61]. Additionally, she was not concerned with the cosmetic outcome (Figure 7).

It is well established that acromion and scapular spine fractures following RTSA can result in significant functional impairment. In a study of 4,125 shoulders treated with a single design of reverse shoulder prosthesis, Routman et al. [23] found that 61 shoulders (1.5%) had radiographically identified acromion and scapular fractures (no clavicle stress fractures occurred). The patients with acromion and scapular fractures had significantly lower outcome scores and less clinical improvement compared to those without fractures. Additionally, 36.4% of the patients with fractures reported being either “worse” or “unchanged” relative to their pre-operative condition, compared with only 8.1% of patients without fracture. These substantial differences highlight the severe consequences of acromion and scapular fractures after RTSA. Our patient’s case, along with the case descriptions of the five reported patients (Table 3) that had clavicle stress fractures in association with RTSA, suggests that these fractures may also cause significant impairment. This highlights the need for more effective methods to prevent and treat stress fractures in these locations.

It is also vitally important to develop pre-operative measures to reduce the risk of stress fractures after shoulder arthroplasty, specifically in patients with poor bone quality. The prevalence of osteopenia or osteoporosis in patients undergoing RTSA is high, with one study reporting 26% of 17,287 patients having these diagnoses at the time of RTSA [62]. Another study reported that 20% of 66 patients (41 men, 25 women) aged >50 years were diagnosed before undergoing RTSA or anatomic TSA [63]. That study speculated that the true prevalence is likely greater; this is because not all cases of bone mineral density (BMD) diseases are diagnosed pre-operatively. In total hip and knee arthroplasty patients, osteoporosis is associated with aseptic loosening, malpositioning of implanted components, and subsidence of implanted components, among other complications [64]. Casp et al. [62] found that osteoporotic patients who underwent RTSA or anatomic TSA were at an increased risk of periprosthetic fractures and revision surgery within two years of these procedures. (These authors used a diagnostic code that would include stress fractures but did not specify whether stress/insufficiency/fragility types of fractures were excluded.) These complications are common to all prosthesis designs used in RTSA [23]. As the use of RTSA expands, the incidence of stress fractures in association with this procedure will increase unless steps are taken to minimize their occurrence.

Although our patient’s pre-operative radiographs (Figure 1) showed clear evidence of osteoporosis, we unfortunately did not perform any workup or treatment for this. Several studies describe the use of pre-operative measurements that are aimed at mitigating the occurrence of periprosthetic and stress fractures after shoulder arthroplasty [63-65]. For example, Hayden et al. [65] describe an algorithm that involves the use of pre-operative computed tomography (CT) scans to obtain BMD measurements of the anatomic neck of the proximal humerus, along with FRAX scores to predict patients with poor bone quality prior to shoulder arthroplasty. Cronin et al. [63] also describe a method that uses CT scanning to assess BMD of the proximal humerus and glenoid prior to shoulder arthroplasty. Early detection of low BMD would allow for early treatment and possible alteration of surgical technique [66].

Despite the fact that bisphosphonates have been used successfully for decades as a treatment for osteoporosis, their inhibition of bone growth/renewal can slow some aspects of fracture healing (e.g., secondary osteon remodeling) [67-69]. Mai et al. [70] have shown that treatment with bisphosphonates for osteoporosis before shoulder arthroplasty was a significant risk factor for intraoperative fracture and one-year post-operative complications, including aseptic loosening, scapular notching, and periprosthetic traumatic and stress fractures. This is thought to be related to long-term suppression of bone remodeling, resulting in the accumulation of bone microdamage and increased mean tissue age, which ultimately can worsen skeletal fragility [71]. This problem can be obviated by the use of non-bisphosphonate treatment options, including anabolic/osteogenic agents such as teriparatide (an injectable low molecular weight parathyroid hormone), selective estrogen receptor modulators, and antiresorptive agents that inhibit RANK-L, such as denosumab [64,70,72]. Teriparatide was the desired treatment for our patient because it increases formation of bone tissue by directly stimulating bone formation within active remodeling sites and previously inactive surfaces of the bone, while also increasing initiation of the new remodeling sites [73,74]. However, our patient was not able to obtain this treatment due to its relatively high cost. This issue also needs to be rectified in order to optimize treatment of patients with deficient bone quality/quantity prior to joint arthroplasty.

## Conclusions

To our knowledge, this is the first reported case describing a patient with acromion and clavicle stress fractures occurring in association with ipsilateral RTSA. We report this unique case and highlight the generally positive outcome for our patient, despite the clavicle fracture’s failure to heal with bone-growth stimulator treatment. Additionally, this case emphasizes the importance of pre-operative bone density and health screenings to reduce the risk of stress fractures after RTSA.
